# The Bacteriological Diagnosis of Diphtheria

**Published:** 1899-06-24

**Authors:** 


### THE BACTERIOLOGICAL DIAGNOSIS OF DIPHTHERIA.

In a very careful article on the diagnosis of certain notifiable diseases, Dr. Foord Caiger,[1] medical superintendent of the South-Western Fever Hospital, lays down the position which may at present be assigned to the bacteriological test in the diagnosis of diphtheria. It is very necessary that the limitations of and disabilities which attach to this test should be thoroughly understood, otherwise very erroneous inferences may easily be drawn from the results obtained. In the first place, we occasionally come across a typical and even fatal attack, with distinctive clinical appearances of diphtheria, in which repeated and careful examination may fail to show a single diphtheria bacillus, while on the other hand characteristic and virulent bacilli may not infrequently be found in the throat secretions of perfectly healthy persons, especially if they have been in contact with cases of diphtheria. Now, whatever we may say about the infectivity of such persons, one would certainly not be justified in saying that they are suffering from diphtheria. The bacilli have also been found in a large proportion of cases of scarlet fever, very few of which presented symptoms suggesting the existence of diphtheria. Again, they have been found in great profusion in the rhinorrhœa which so often follows scarlet fever. Another and an even stronger argument against the infallibility of the bacteriological test is the wide difference of opinion which exists amongst eminent bacteriologists as to what is and what is not a diphtheria bacillus. Under such circumstances one may well ask, What is the practical value of such a test in actual practice? Dr. Caiger says, "A negative result after careful examination of the material taken from the throat, and of the culture derived from it, on at least two occasions, may be held practically to exclude diphtheria. A positive result after examination of a throat presenting a definite pellicular exudation, or from the larynx without visible exudation on the fauces, may be regarded as sufficient evidence of diphtheria. The presence of short bacilli (suggesting diphtheria bacilli) in the nasal discharge alone, even though attended with isolated spots of exudation on the tonsils, cannot be held to be distinctive, unless confirmed by inoculation into animals."

[1] Lancet, June 17.

